# An exploration of learning needs: identifying knowledge deficits among hospitalized adults with heart failure

**DOI:** 10.3934/publichealth.2019.3.248

**Published:** 2019-08-02

**Authors:** Erika Raines, Sabrina L. Dickey

**Affiliations:** College of Nursing, Florida State University, Tallahassee, Florida, United States

**Keywords:** chronic heart failure, congestive heart failure, heart failure, self-care, learning needs, knowledge

## Abstract

The current study examined the learning needs of hospitalized patients with chronic heart failure (CHF) by identifying their current CHF self-care behaviors and knowledge levels, while identifying relationships between clinical variables, and their learning needs. A descriptive, cross-sectional design was utilized to examine a convenience sample of 42 hospitalized patients with CHF. Self-care behaviors and knowledge levels were measured using the Self Care of Heart Failure Index V. 6.2, and the Japanese Heart Failure Knowledge Scale. Descriptive statistics were used to describe the learning needs and sociodemographic data and Pearson product moment correlation examined relationships between the learning needs and clinical variables. The mean scores of self-care were 38.6% (maintenance), 41.6% (management) and 17.8% (confidence). The participant's mean knowledge level score was 74.9%. Multiple CHF hospital readmissions had a significant negative correlation with CHF knowledge scores (*r* = −0.358, *p* < 0.05), suggesting the lower the knowledge score, the higher the prevalence of CHF readmissions. There were significant positive correlations between self-care maintenance (r = 0.525, *p* < 0.05), management (r = 0.435, *p* < 0.05), confidence (r = 0.366, *p* < 0.05), knowledge level (r = 0.752, *p* < 0.05) and not living alone. Thus, indicating that living with family support is correlated with better self-care and greater knowledge. The presence of multiple comorbidities revealed significant positive correlations (*p* < 0.05) with self-care scores (maintenance [r = 0.391], management [r = 0.438], and confidence r = 0.504), indicating a higher number of comorbidities, yielded lower self-care behaviors. Findings revealed poor self-care behaviors in all three areas (maintenance, management, confidence). These findings suggested that patients had difficulty implementing knowledge into self-care. By identifying knowledge deficits and contributing factors to suboptimal self-care, the chronic care model can be used as a guideline for ideal CHF education and management. Improving self-care behaviors can be obtained by initiating an active engagement between health care teams and the patient.

## 1. Introduction

Heart Failure with reduced Ejection Fraction (HFrEF) or chronic heart failure (CHF) results from a structural or functional impairment of ventricular filling or ejection of blood. Clinical complications of CHF include, but are not limited to dyspnea, fatigue or fluid retention (edema) and can result from disorders of the pericardium, myocardium, endocardium, heart valves, or great vessels with most patients suffering from impaired left ventricular (LV) myocardial function [1]. Chronic heart failure is a serious health concern that affects 5.7 million adults in the United States while also producing multiple implications to the healthcare system [2]. An estimated $30.7 billion dollars is spent each year towards the treatment of CHF, including total cost of health care services, medications, and missed days of work [3]. Compounding the economic burden of CHF, research has revealed the substantial problem of hospital readmissions with an approximated 25% of CHF patients re-hospitalized within 30 days of discharge [4]–[6]. In addition, an average of 50% of those diagnosed with CHF die within 5 years of diagnosis [5].

### 1.1. Heart failure and the impact on society

Heart failure is a progressive syndrome characterized by increased mortality, frequent hospitalizations, and a multifaceted therapeutic regimen [7]. The lifetime risk of developing CHF in Americans over the age of 40 is 20%. The incidence of CHF increases with age, rising from an estimated 20 per 1,000 individuals aged 65 to 69, to greater than 80 per 1,000 individuals among those over the age of 85. Additionally, the black population has the highest risk of CHF, while also having a greater five-year mortality rate, compared to Whites [8]. Chronic heart failure is cited in one in nine deaths in the United States with rates of cardiovascular-related deaths greatest in those previously hospitalized for CHF [2].

Chronic heart failure is a burdensome disease process, projected to increase by 8 million by the year 2030 [10]. Additionally, CHF is expected to cause an increase in hospital readmissions over the next 10 years, and the disease itself is due to several factors. One of those factors involves the large number of prescribed medications which requires multifaceted daily dosing schedules. Furthermore, adult patients with CHF are likely suffering from multiple comorbidities such as coronary artery disease (CAD), diabetes, or hyperlipidemia [8]. Research indicates that management of CHF goes beyond medical management and requires several lifestyle adjustments and nonpharmacological interventions in order to prevent disease progression or exacerbation. Such interventions include diet modification, exercise training, consistent monitoring of weight, close examination of signs and symptoms, and prompt reporting of these signs and symptoms [5]. These practices illustrate the concept of self-care, which is defined as behaviors to promote health and well-being while managing illness [9]. The promotion of self-care, specialized outpatient education, and proper follow-up among CHF will be an integral aspect for a person's ability to manage his or her own disease, symptoms, and treatment to improve their overall quality of life [10],[11].

### 1.2. Hospital readmissions

Under the Hospital Readmission Reduction Program of the Patient Affordable Care Act, hospitals with CHF readmission rates above the national average are receiving reductions or penalties in Medicare reimbursements [12]–[14]. Because of this, the Centers for Medicare and Medicaid Services (CMS) have made 30-day hospital readmission rates a quality indicator with a focus on improving disease management in the specified areas of acute myocardial infarction, pneumonia, and CHF [5],[15]. With the consequence of readmission penalties, healthcare systems are now challenged to prevent readmissions [12],[13]. However, the rising number of patients with CHF and the amount of hospitalizations is likely to raise considerably regardless of the financial penalties inflicted by Medicare for all-cause readmission within 30 days of discharge [16].

An estimated 50% of CHF hospital readmissions are considered preventable, related to poor adherence with self-care recommendations, and is recognized as the contributing factor in readmissions [8]. Research shows that patients who are proactively involved in the management of their disease (self-care) and possess the ability and knowledge level to adhere to specialized medical regimens have better survival outcomes and reduced hospital admissions [17].

### 1.3. Current guidelines

Practice guidelines have become a large part of the clinical environment and have become more rather than less universal. A more patient-centered validation for the development of guidelines has become especially common for the potentially fatal syndrome of CHF [18]. In June 2013, the American College of Cardiology Foundation (ACCF) and the American Heart Association (AHA) published an updated “ACCF/AHA Guideline for the Management of Heart Failure: A Report of the American College of Cardiology Foundation/American Heart Association Task Force on Practice Guidelines”. The updated guidelines were developed to assist providers in decision making regarding the diagnosis and treatment of CHF. According to this report, every patient with CHF should have a clear, comprehensive, and evidence-based plan of care. Ultimately, an inclusive plan of care should be provided for successful patient self-care [19]. Organizations, such as the Heart Failure Society of America (HFSA) developed inclusive guidelines in 2010, which use a team approach, screen patients for literacy, psychological state, culture, and access to social and financial resources in order to optimize CHF education and self-care [18].

### 1.4. Importance of self-care

According to Riegel et al. self-care is defined as “a naturalistic decision-making process that influences actions that maintain physiologic stability, facilitate the perception of symptoms, and direct the management of those symptoms” (p. 226) [20]. The authors also developed and revised a situation-specific theory of CHF self-care, which consists of a three-step process 1) maintenance; 2) symptom perception; and 3) management. The three-step process encompasses issues related to medication adherence, diet modifications, recognition of physical sensations and the interpretation of these sensations, and the response to the symptoms at the time they occur [20].

Regular symptom monitoring has been shown to enable the performance of satisfactory self-care management, which may contribute to a decrease in unnecessary hospitalizations for CHF [21]. However, patient nonadherence to suggested self-care activities is common and self-care is fundamental to the maintenance of improved physiologic solidity among clients with CHF [22]. Better adherence to self-care management of CHF is associated with improved health status which highlights the need for further clinical research regarding self-care to optimize CHF outcomes [8].

### 1.5. Chronic Heart failure education

Education for CHF patients is an intricate and multifaceted process with no gold-standard approach identified to date [23]. Traditional CHF education supplied as part of hospital-based discharge teaching aims to take advantage of a “teachable moment” after an event, however improvement in self-care using this approach is often lost after hospitalization [24]. Even though considerable resources for education are accessible and related to CHF, the bulk of patients do not retain or execute this knowledge into the management of their disease [25]. Patient education is often inadequate due to time constraints and patient fatigue during hospitalization. Given the breadth of education required, it is vital to prioritize the delivery of education that includes the perceived learning needs of patients [26]. Well-written educational materials or workbooks allow patients to learn at their own pace and can be used as an ongoing reference [23]. Multimedia educational interventions have also shown promise in reducing unplanned CHF readmissions when used in combination with a written manual [27]. Ultimately, the use of multimedia and community-based interventions have far-reaching implications for helping CHF patients improve their self-care [24],[27].

### 1.6. Management of heart failure

The importance of post-discharge follow-up care following a hospital admission related to CHF is crucial in ensuring that vulnerable populations receive proper care [28]. Strategies that focus on CHF management and improvement in CHF readmission rates include inpatient approaches, such as, clinical pathways or specialized models, namely disease management programs (DMP). Clinical pathways, nurse navigator teams and disease management programs are different strategies to reduce CHF readmissions, however providing patients with CHF education seemed to be a common element in the strategies. In fact, literature indicated that nurse educator delivered teaching at the time of hospital discharge may result in enhanced clinical outcomes, improved self-care, and reduced cost of care in addition to a decreased risk of hospital readmission or death. Additionally, the assimilation of a pharmacist in discharge support has also proved to be advantageous in reducing 30-day mortality and CHF-related readmissions [28],[29]. Similar results reported a significant increase in education and follow up among CHF patients, which yielded a 30 day all cause readmission rate that was 8% lower compared to patients not involved in a CHF program [30].

Disease management programs are outpatient-focused approaches to minimize hospital readmissions by providing a multidisciplinary approach to care for CHF patients [12]. Examples of DMP's include outpatient clinic interventions, home care (home health), structured telephone support, and tele monitoring. The use of CHF clinics demonstrated significant reductions in readmissions [31]. Multidisciplinary CHF clinics focused on post-hospitalization follow up were associated with a substantial reduction in both 30 and 90-day CHF readmissions [32],[33]. Results from a nurse-led heart failure clinic reported CHF patients who attended the clinic over a six-month period had lower mortality rates and a significant reduction in their systolic and diastolic blood pressure compared to those who did not visit the clinic [34]. Similar results were indicated in a DMP which utilized an explanatory workbook and recurrent telephone contact over one year, wherein the readmission rate was lower for CHF patients compared to usual care at 16 months (26.3% vs. 31%) [35].

### 1.7. Patient-level barriers

Regardless of the benefits of self-care, CHF patients, particularly among the elderly, face various difficulties and barriers in performing optimum self-care behaviors. These difficulties and barriers for performing self-care consist of misconceptions, lack of knowledge, age, race, ethnicity, socioeconomic status, and the limited amount of time for health care providers to deliver health education [36]. These factors were associated with increased readmission rates and poor outcomes in patient education and teaching [37]–[39]. Patient perceptions of instructions often differ which causes an inability of patients to fully understand the instructions provided by his or her doctor [40]. Regarding age, the presence of other comorbidities and older adults (age 73 or older) have shown to have decreased sensory nerve perception and difficulty distinguishing and interpreting symptoms, such as shortness of breath [22]. Psychosocial and psychological factors, such as depression and anxiety and lack of support were associated with poor self-care among CHF patients [40]. Therefore, a multitude of factors must be considered upon examining the readmission rates among CHF patients and their knowledge deficits.

### 1.8. Theoretical framework

The chronic care model is a guide to improve chronic illness management and identifies six key components: 1) community resources and policies; 2) self-management support; 3) health care organization; 4) delivery system design; 5) decision support; and 6) clinical information systems. In order to improve chronic care, provider organizations require linkages to community-based resources and should have structured goals. Furthermore, providers must view chronic care as a priority and detach acute care from planned management of chronic diseases [41].

Chronic heart failure management requires the use of combined systems within the healthcare realm as well as active patient participation. The chronic care model blends patient self-management (self-care) support with health system change to accomplish productive interactions between a proactive health care team and an engaged patient [42]. A multidisciplinary approach to self-care (self-management), along with close monitoring of clinical constraints may have beneficial effects on the outcome of patients with CHF [43]. By identifying knowledge deficits and contributing factors to suboptimal self-care, the chronic care model can be used as a guideline for ideal CHF education and management.

### 1.9. Purpose and aims

The purpose of this study was to examine the learning needs of hospitalized patients with CHF by identifying their current CHF self-care behaviors and knowledge levels in addition to identifying relationships between patient learning needs and select clinical variables (sociodemographic and health history data). The current study addressed the following clinical questions: 1) what are the self-care behaviors and knowledge levels among adult patients with CHF on an inpatient cardiac unit at an acute care hospital? and 2) what relationships exist between the patient's learning needs and select clinical variables (sociodemographic and health history data)?

## 2. Materials and methods

### 2.1. Design, setting, and sample

A descriptive cross-sectional design was used in this study. The use of three questionnaires were used to assess the learning needs of participants. Approval for this study was granted through the hospital research committee where the study was performed, as well as the University's Human Subjects Committee.

A convenience sample of 42 patients with a history of CHF were recruited. The study was carried out on a 40-bed adult cardiac unit in an acute care hospital located in Southwest Florida from January 2018 to March 2018. Patients were recruited by scanning the unit census and accessing each patient's medical record individually, searching for individuals with a history of CHF. Inclusion criteria were participants with a clinical diagnosis of CHF, displayed as “Heart Failure”, “Chronic Heart Failure”, “HF” or “CHF” documented in their medical record by a physician, nurse practitioner, or physician assistant. To avoid surveying patients with new onset CHF, or those being ruled out for the disease, the diagnosis would have to be documented in a record from a previous hospital visit. Additional inclusion criteria for this study included: 1) age 18 years or older and 2) able to read, write, and speak English. Individuals were excluded if they were currently enrolled in any kind of formal CHF program.

### 2.2. Measures

Sociodemographic and health history data were obtained using a 22-item questionnaire generated with influence from the *Behavioral Risk Factor Surveillance System* (*BRFSS*). The BRFSS is used by the Centers for Disease Control and Prevention to collect state data from residents in the United States regarding their health-related risk behaviors, chronic health conditions, and use of preventive services [44]. Multiple areas of interest were explored, such as income level, history of hospital admissions related to CHF, whether they lived alone or not, and the presence of comorbid disorders, among others.

*The Self-Care Heart Failure Index.* The SCHFI V 6.2 is a 22-item instrument comprised of three scales that measure theoretically derived components of CHF self-care: maintenance, management, and confidence [45]. The self-care maintenance scale assesses symptom monitoring and adherent behaviors implemented to prevent CHF exacerbation. The self-care management scale calculates the patients' ability to recognize symptoms as they occur, treatment interventions in response to symptoms, and treatment evaluation. The self-care confidence scale measures the patient's perceived capacity to engage in each phase of the self-care process [46]. Each scale utilizes a 4-point Likert scale in a self-report response format: 1 (never or rarely), 2 (sometimes), 3 (frequently), and 4 (always or daily). A specific scoring algorithm was utilized to produce a standardized score from 0 to 100; higher scores indicate better self-care. When the SCHFI V.6.2 update was published, Cronbach alpha was reported to be .55 for self-care maintenance, .60 for self-care management, and .83 for self-care confidence [45]. In a recent study, the SCHFI v 6.2 was found to have evidence of construct validity, contrasting groups' validity, internal consistency, and test-retest reliability, which supported its continued use in research [46].

*Japanese Heart Failure Knowledge Scale.* The Japanese Heart Failure Knowledge Scale is a 15-item true or false survey that evaluates the knowledge of patients regarding CHF pathophysiology, symptoms, treatment, and self-care. This tool was chosen among numerous pre-existing CHF knowledge assessment tools due to its acceptable validity and reliability, demonstrated in a recent study, with Cronbach's alpha at 0.79 [47]. Scores for each item were summed to produce a range, from 0 to 15, and were calculated into a percentage out of 100. Higher scores denoted greater knowledge about CHF [48].

### 2.3. Procedure

The principal investigator (PI) identified all eligible participants through an electronic chart review and approached patients in person during their inpatient stay. Potential participants were provided with a study information packet which included the following items: an explanation of the purpose of the study, informed consent, and questionnaires. The potential subjects were given the opportunity to ask questions at any time. If the potential participant agreed to participate in the study, an informed consent was obtained and questionnaires were completed. After participants completed the three questionnaires, the entire study packet was returned to the PI and kept in a locked and protected location. All study packets were collected after the questionnaires were completed and the participants were not allowed to keep them overnight. The duration of each data collection session ranged between 15 to 45 minutes. No identifiable data was recorded in the questionnaires and participation was voluntary. No data from this study was part of the patient's permanent medical record. Additionally, no incentive was provided to participants in this study.

### 2.4. Data analysis

The Statistical Package for the Social Sciences (SPSS) version 24.0 (IBM Inc., Armonk, NY, USA), was used for descriptive and statistical analysis. Descriptive statistics were used to summarize and describe the learning needs and sociodemographic data of the participants. Pearson product moment correlation coefficient was utilized to assess relationships between learning needs and sociodemographic and health history data. The level of significance was set at *p* < 0.05 for the statistical data.

## 3. Results

### 3.1. Demographics

There were 77 patients approached throughout the data collection time for participation in the study, which yielded a total of 42 patients that participated. The average age of participants was 70.83 (SD = 10.070) with a minimum age of 43 and a maximum age of 93 years. Most of the participants were male (57.1%), non-Hispanic (97.6%), White (76.2%), divorced (40.5%), with a high school degree or above (92.8%), retired (73.8%), and earned less than $50,000 a year (64.3%). Demographic results are presented in Table 1.

**Table 1.** Demographics.

Characteristic	N	Percent (%)
Gender		
Male	24	57.1
Female	18	42.9
Ethnicity		
Hispanic, Latino or Spanish	1	2.4
Non-Hispanic	41	97.6
Race		
White	32	76.2
Black or African American	10	23.8
Marital Status		
Married	12	28.6
Divorced	17	40.5
Widowed	8	19.0
Separated	2	4.8
Never Married	3	7.1
Education		
Some High School	3	7.1
High School Graduate	16	38.1
Some College	10	23.8
College Graduate	13	31.0
Employment		
Employed	1	2.4
Out of work > 1 year	10	23.8
Retired	31	73.8
Income		
< 10K	1	2.4
< 15K	1	2.4
< 20K	5	11.9
< 25K	8	19.0
< 35K	1	2.4
< 50K	11	26.2
< 75K	7	16.7
> 75K	8	19.0

### 3.2. Other clinical variables of interest

Healthcare access. Most participants had some form of healthcare (81%), Medicare (69%). Additionally, most participants had more than one healthcare provider (81%), had seen a provider in the last 12 months for a routine check-up (88.1%), and experienced no delays in receiving medical care anytime within the past one year (71.4%). The most common reason for delayed medical care was lack of transportation (23.8%) and cost caused 26.2% of participants to delay medical care in the past 12 months. Table 2 reflects the results from the other clinical variables of interest which was healthcare access.

**Table 2.** Healthcare access.

Character	N	Percentage (%)
Do you have any kind of health care coverage, including health insurance, prepaid plans such as HMOs, or government plans such as Medicare, or Indian Health Service?		
Yes	34	81.0
No	8	19.0
Do you have Medicare?		
Yes	29	69.0
No	13	31.0
Do you have one person you think of as your personal doctor or health care provider?		
Yes, only one	1	2.4
More than one	34	81.0
No	7	16.7
Was there a time in the past 12 months when you needed to see a doctor but could not because of cost?		
Yes	11	26.2
No	31	73.8
About how long has it been since you last visited a doctor for a routine checkup?		
< 12 months	37	88.1
1 year < 2 years	2	4.8
2 years < 5 years	2	4.8
5 years or more	1	2.4
Have you delayed getting needed medical care for any of the following reasons in the past 12 months?		
Could not get through on the telephone	1	2.4
Could not get an appointment soon enough	1	2.4
You did not have transportation	10	23.8
No I did not delay in getting medical c are	30	71.4

*Health history.* The most common response regarding a rating of their general health was stated as only “fair” (57.1%) with the remainder of participants reporting it as “good” (42.8%). Most participants were told they had CHF for less than 5 years (59.5%) and many did not know how long they had the disease (26.2%). Less than half of the participants were hospitalized within the past 30 days for illness related to CHF, however, 64.3% of participants were hospitalized 1 time or more for CHF decompensation within the past year. The highest reported comorbidities were hypertension (95.2%) and hyperlipidemia (71.4%) with most participants reporting 3 or more comorbidities (88.1%). Additionally, more than half of the participants reported living alone (57.1%). The participants' health history characteristics are presented in Table 3.

**Table 3.** Health History.

Characteristic	N	Percentage (%)
Would you say that in general your health is		
Good	18	42.8
Fair	24	57.1
How long have you been told you have Heart Failure or Congestive Heart Failure?		
< 1 year	11	26.2
2–5 years	14	33.3
6–10 years	4	9.5
11–20 years	2	4.8
Not sure	11	26.2
Have you been hospitalized within the last 30 days for illness related to Heart Failure or Congestive Heart Failure?		
Yes	19	45.2
No	23	54.8
How many times in the past 1 year have you been hospitalized for illness related to Heart Failure or Congestive Heart Failure?		
None	12	28.6
1 time	12	28.6
2 times	7	16.7
3–5 times	8	19.0
Not sure	3	7.1
Do you currently, or have you ever had in the past, any of the following?		
Diabetes	24	57.1
Hypertension	40	95.2
Coronary Artery Disease	23	54.8
Heart Attack	22	52.4
Hyperlipidemia	30	71.4
Kidney Disease	12	28.6
COPD	10	23.8
Do you live alone?		
Yes	24	57.1
No	18	42.9

### 3.3. Learning needs of patients with heart failure

Descriptive statistics such as percentages, means, and standard deviation were used to characterize the learning needs (self-care behaviors and knowledge level) of participants. Both self-care behaviors and knowledge level were scored on a scale from 0 to 100 (%). Each scale of self-care (maintenance, management, and confidence) were scored individually. A higher mean was indicative of increased competency of self-care management skill and knowledge level. Among the 3 self-care scales, participants performed the highest in self-care management (41.6%), second highest in self-care maintenance (38.6%), and lowest in self-care confidence (17.7%). The average score of CHF knowledge among the participants was 74.9%. An incidental finding that was obtained when screening individuals for participation in the study was the number who were unaware they had “Heart Failure, or “Chronic Heart Failure”. Of the 42 participants surveyed, 14 individuals (33%) could not certainly say they had the diagnosis, even though it was documented in their medical history record. Several participants stated they “think” CHF was what they had, confirming they had “heart problems”, but did not recognize the term “Heart Failure” or “Congestive Heart Failure” specifically. Often, after the words “swelling”, or “shortness of breath” were spoken to describe their disease process during the pre-study interview, the patient could then confirm the diagnosis. The self-care measures of central tendency are displayed in Table 4.

Specific questions from the SCHFI were examined regarding the self-care concepts of maintenance, management, and confidence. Regarding self-care maintenance, the largest response was participants sometimes asked for low salt items when eating out or visiting others. The category of self-care management that yielded the largest response was participants indicating they were not likely to reduce fluid intake, 79%. Lastly, self-care confidence yielded most responses as somewhat confident under the options of doing something that will relieve your symptoms and evaluating the importance of symptoms as 57% for both. A listing of the responses is presented in Table 5. In addition, questions from the Japanese Heart Failure Knowledge Scale were analyzed individually and are displayed in Table 6. The CHF knowledge level questions were true or false, therefore the percentage wrong can be easily inferred.

**Table 4.** Self-care measures of central tendency.

	Self-Care Maintenance	Self-Care Management	Self-Care Confidence	HF Knowledge
n (Valid)	42	42	42	42
Mean	38.56	41.58	17.77	74.92
Std. Deviation	12.90	24.03	11.97	11.09
Range	59.99	80.99	53.33	40.00
Minimum	16.67	10.00	13.33	53.33
Maximum	76.66	90.99	66.66	93.33

**Table 5.** SCHFI results.

SECTION A: MAINTENANCE				
How routinely do you do the following?	Never/Rarely	Sometimes	Frequently	Always/Daily
Weigh yourself	40% (n = 17)	43% (n = 18)	2% (n = 1)	14% (n = 6)
Do some physical activity	40% (n = 17)	50% (n = 21)	7% (n = 3)	2% (n = 1)
Keep doctor or nurse appointments	0% (n = 0)	7% (n = 3)	48% (n = 20)	45% (n = 19)
Eat a low salt diet	24% (n = 10)	55% (n = 23)	17% (n = 7)	5% (n = 2)
Exercise for 30 minutes	79% (n = 33)	14% (n = 6)	5% (n = 2)	2% (n = 1)
Forget to take one of your medicines	57% (n = 24)	43% (n = 18)	0% (n = 0)	0% (n = 0)
Ask for low salt items when eating out or visiting others	36% (n = 15)	64% (n = 27)	0% (n = 0)	2% (n = 1)
Use a system (pill box, reminders) to help remember your medicines	14% (n = 6)	5% (n = 2)	24% (n = 10)	57% (n = 24)

SECTION B: MANAGEMENT					
If you had trouble breathing or ankle swelling in the past month……	Did not recognize	Not Quickly	Somewhat Quickly	Quickly	Very Quickly
How quickly did you recognize it as a symptom of heart failure?	24% (n = 10)	21% (n = 9)	24% (n = 10)	24% (n = 10)	7% (n = 3)
How likely are you to try one of these remedies?		Not Likely	Somewhat Likely	Likely	Very Likely
Reduce salt in your diet		71% (n=30)	26% (n=11)	2% (n = 1)	0% (n = 0)
Reduce your fluid intake		79% (n = 33)	12% (n = 5)	7% (n = 3)	2% (n = 1)
Take an extra water pill		57% (n = 24)	12% (n=5)	29% (n = 12)	2% (n = 1)
Call your doctor or nurse for guidance		34% (n = 10)	10% (n = 4)	43% (n = 18)	24% (n = 10)
How sure were you that the remedy helped or did not help?	Did not try anything	Not sure	Somewhat Sure	Sure	Very Sure
How sure were you that the remedy helped or did not help?	24% (n = 10)	36% (n = 15)	26% (n = 11)	10% (n = 4)	5% (n = 2)

SECTION C: CONFIDENCE				
In general, how confident are you that you can:	Not Confident	Somewhat Confident	Very Confident	Extremely Confident
Keep yourself free of heart failure symptoms	14% (n = 6)	71% (n = 30)	14% (n = 6)	0% (n = 0)
Follow the treatment advice you've been given	14% (n = 6)	29% (n = 12)	50% (n = 21)	7% (n = 3)
Evaluate the importance of your symptoms	33% (n = 14)	57% (n = 24)	7% (n = 3)	2% (n = 1)
Evaluate how well a remedy works	36% (n = 15)	53% (n = 22)	10% (n = 4)	2% (n = 1)

### 3.4. Relationship between Learning Needs and Clinical Characteristics

The mean scores of the learning needs (self-care behaviors and knowledge level) were taken and measured for associations with the participant's characteristics using Pearson's correlation. The three-specific variable of interests chosen to evaluate for associations with the learning needs were: 1) history of any CHF admissions within the past year; 2) whether the participant lives alone; and 3) the presence of three or more comorbidities.

There was a significant negative correlation between CHF knowledge scores (*r* = −0.358, *p* = 0.020) and CHF admissions within the past year. In this case, the lower the CHF knowledge score, the higher the number of CHF admissions. There were no significant correlations between self-care maintenance, management, or confidence and having any CHF admissions within the past year.

**Table 6.** Knowledge Results.

HF Knowledge	
(Using The Japanese Heart Failure Knowledge Tool)	% Correct
Exchange of oxygen and carbon dioxide occurs in the heart	55% (n = 23)
Heart Failure is a condition in which the heart is not able to pump blood through the body in sufficient amounts	95% (n = 40)
Difficulty breathing and shortness of breath are symptoms of Heart Failure	100% (n = 42)
One of the symptoms when the lungs become congested with fluid is shortness of breath	93% (n = 39)
Some patients with severe Heart Failure become breathless when they lie flat and feel much better when they sit up	79% (n = 33)
Short-term weight gain is one of the signs of worsening Heart Failure	55% (n = 23)
Overwork and stress sometimes causes Heart Failure to get worse	86% (n = 36)
Sodium causes water retention	100% (n = 42)
Diuretics remove fluids from the body	88% (n = 37)
Heart Failure patients are discouraged from taking medications without food	52% (n = 22)
Heart Failure patients had better drink more water than healthy people	36% (n = 15)
Heart Failure patients had better take a high-salt diet	100% (n = 42)
Smoking is good for patients with Heart Failure because it promotes the circulation of blood	100% (n = 42)
Heart Failure patients should not perform exercise regardless of their severity of Heart Failure	43% (n = 18)
Heart Failure patients better take a hot bath to promote blood circulation	88% (n = 37)

As seen in Table 7, there were significant positive associations between self-care maintenance (r = 0.525, *p*< 0.05), management (r = 0.435, *p* < 0.05), confidence (r = 0.366, *p* < 0.05), knowledge level (r = 0.752, *p* < 0.05), and *not* living alone. Thus, not living alone, i.e. living with a spouse or family member (family support) was associated with increased scores of self-care behaviors (maintenance, management, and confidence), and CHF knowledge.

Having three or more comorbidities present revealed significant positive correlations (*p* < 0.05) with self-care scores (maintenance [r = 0.391], management [r = 0.438], and confidence r = 0.504]). There was a non-significant relationship with CHF knowledge (*p* > 0.05, r = 0.277). These results indicated that the presence of three of comorbidities was associated with lower self-care scores.

**Table 7.** Pearson's correlation.

Variable	Self-Care Maintenance	Self-Care Management	Self-Care Confidence	HF Knowledge
HF Admissions	−0.157	−0.177	−0.200	−0.358*
Living alone	0.525*	0.435*	0.366*	0.752
Presence of three or more comorbidities	0.391*	0.438*	0.504*	0.277

## 4. Discussion

This study sought to examine the present learning needs of hospitalized adult patients with CHF. Findings revealed poor self-care behaviors in all three areas (maintenance, management, and confidence) as evidenced by low self-care means. It is interesting to note that the higher level of elderly participants who were also retired resulted in a higher number which had some type of health insurance (81%). This implies that poor self-care is probably not related to the lack of health insurance among participants in this study. All participants (100%) reported they were knowledgeable regarding shortness of breath or difficulty breathing as an indication of worsening CHF. This finding contrasts with studies that reported older adults (age 73 or older) had more difficulty distinguishing and interpreting symptoms such as shortness of breath [22]. Furthermore, in two literature reviews results indicated newly diagnosed and experienced CHF patients had difficulty recognizing symptoms [38] and there was knowledge of CHF symptoms, but it was not at an adequate level [49]. The mean score of CHF knowledge among participants was significantly higher than the mean scores of self-care behaviors, but had room for improvement. These findings indicate that patient's may understand their disease process but have difficulty implementing their knowledge into self-care. This supports previous literature which indicated that patients with CHF expressed difficulty with translating knowledge into understanding how to engage in self-care activities and behaviors [50]. The incidental finding regarding a lack of awareness for their CHF diagnosis was supported by studies in which participants had minimal knowledge and were unaware they were diagnosed with CHF [37],[49]. Findings suggested the need for increased teaching when defining the disease process and differentiating it from other heart-related problems such as heart attack or arrhythmia. Additionally, the chronic care model's components of self-management, health care organization, delivery system design, decision support, and clinical information system aligned with the study's aim. As previously mentioned the aim was to examine self-care knowledge and behaviors among hospitalized CHF patients and the relationships among the patient's learning needs and various sociodemographic and health history data. Evaluation and promotion of self-care skills is crucial in executing a successful management system for CHF patients [51].

### 4.1. Learning needs

Although a portion of self-care behavior scores were less than ideal, the findings align with previous studies wherein self-care scores in the areas of medication adherence, exercise, weight, diet (sodium restriction), and symptom recognition were less than satisfactory [38],[49]. Daily weights are an essential element in the self-care of CHF which had a low adherence among participants. Among CHF patients, literature has indicated a lack of understanding for obtaining daily weights and non-adherence for obtaining daily weights and recording the weights as recommend by CHF guidelines [21],[49],[52]. However, another factor for not implementing daily weights could be their inability to afford a weight scale. On another note, there were several areas of self-care and knowledge wherein participants reported proficiencies. The study indicated that most participants kept their doctor's appointments, used a pill box or medication reminder, called their healthcare provider to report swelling or shortness of breath, followed advice for treatment, and never forgot to take their medication. These findings are comparable to conclusions in a systematic review which revealed CHF patients reported greatest adherence to keeping follow up appointments and taking medication as prescribed [38].

*Medication compliance.* Medication nonadherence in CHF is usually related to an increased risk of clinical effects like mortality and hospitalization [22]. The results of this study suggested that patients adhered to medication regimens. Consistent with previous literature, adherence was also reported with medications [8]. The ability to identify medication dosage, frequency, reason for taking the medications, reading labels, opening bottles, and distinguishing between their pills are useful in the self-management of medications in the elderly populations [53].

*Fluid intake.* The results from the participant's knowledge of fluid intake indicated a lack of awareness regarding the importance of fluid restriction in the management of their disease. More than half of the participants in the current study reported CHF patients should drink as much water as individuals without CHF and only 9% were likely or very likely to decrease their fluid intake if they experienced shortness of breath or swelling. Similar results from a previous study, indicated 39% of CHF patients discharged from the hospital had premature clinical decompensation due to failure to restrict fluid [54]. Likewise, a study pertaining to the role of education among CHF patients revealed that one third of patients believed they should drink large volumes of fluids [49].

*Physical activity.* Previous studies suggested that most CHF patients had more difficulty adhering to exercise than compared to other areas of self-care [8]. In congruence with previous literature, only 9% of participants in this study reported doing some sort of physical exercise either frequently or daily and 57% of participants believed they should not perform exercise regardless of the severity of their CHF. These findings suggested that many patients may not recognize the importance of physical activity. Nonadherence to exercise was found to be associated with increased risk of CHF readmission and mortality. In fact, according to the AHA and ACC, exercise is a beneficial adjunct to treatment for patients with current or prior symptoms of CHF [22],[45].

*Self-care confidence.* Of the three scales, self-care confidence was the least proficient area of self-care among participants wherein 85% were only somewhat confident, or not confident at all that they could keep themselves free from CHF symptoms. Self-care confidence, in managing CHF, may be impacted by factors associated with older age, being male, marital status, comorbidities, and education to name a few [55],[56]. Descriptive factors in the current study which indicated many of the participants as male, divorced, 70 years of age, only a high school diploma, and several comorbidities mirrored the results of factors related to low self-care confidence scores among individuals with CHF [56]–[58]. However, in previous studies, the self-care confidence mean was above 60%, suggesting moderate self-efficacy for engaging in CHF self-care behaviors [9],[24]. The contrast in results indicated the need for continued examination of factors which influence self-care confidence for engaging in CHF self-care behaviors. Furthermore, it has been shown that patients who have higher self-care confidence scores not only perceive their health as better, but also have a better quality of life [59].

### 4.2. Relationship among heart failure admissions, living alone, and comorbidities

*CHF admissions.* Less than half of participants in this study were readmitted to the hospital for CHF within the past 30 days, however, 57.1% reported being hospitalized due to CHF one or more times within the past year. The results of this study suggested that the less the patient knows about their disease (lower knowledge level), the higher the frequency of CHF hospital admissions. This is consistent with previous literature which found that patients with a history of multiple CHF exacerbations accompanied by frequent hospital visits had lower knowledge levels and often delayed seeking appropriate medical care [31]. Knowledge is a minimum requirement for the behavior changes and self-care strategies needed to avoid frequent hospitalizations and high mortality [60].

*Living alone*. Many patients with CHF live alone and are incapable of accessing established social services [45]. This study revealed a significantly positive correlation between lower self-care and knowledge level scores and not living alone. This correlation indicates that living without a social support system or caregiver would produce lower self-care and knowledge level scores, resulting in sub-optimal disease management. These findings are consistent with previous research that indicated poor self-care has been associated with inadequate family support and supportive environments can provide better adherence to treatment [40]. Caregivers are a vital contribution to the patient's self-care and many patients with CHF depend on the support of their families or social network [61].

*Presence of comorbidities.* Among the population sampled, there appears to be a correlation between poor self-care scores and the presence of comorbidities. This indicated that an increased number of comorbidities made it more difficult for patients to perform efficient self-care. Literature indicated the presence of significant relationships between comorbid conditions and self-care [20],[62]. Additionally, individuals with better-perceived health reported having less comorbidities [59]. Confusion about numerous comorbid conditions and difficulty interpreting healthcare providers' instructions for multiple diseases creates barriers to efficient self-care decision making [45]. This highlights the necessity for identifying the learning needs and knowledge levels of individuals with CHF as well as those with comorbidities.

### 4.3. Limitations

Despite the relevance of the current study in the field of CHF, there were some limitations to be addressed. This study included a small sample size, which restricted the ability to generalize the results to the larger population. It is important to note that all information was self-reported with the exclusion of verifying a CHF diagnosis in the medical record. Therefore, an accurate rate of hospital readmissions and health care access data among others may be under or over reported. The study did not test for literacy or level of cognition which could skew perception and produce inaccurate reporting. Otherwise eligible patients who could not speak, read or write English were not included. There was a relatively high refusal rate (45%) affected by several possible factors such as patients with limited concentration (elderly), altered mental status, high acuity, or general disinterest in the study. Additionally, it is probable that some patients gave imprecise answers due to lack of recall or mild cognitive impairment.

### 4.4. Implications for practice

In order to achieve positive health outcomes among patients with debilitating, chronic diseases such as CHF, it is crucial to first identify the population's learning needs. This study provided important findings on the knowledge deficits among patients with CHF as well as the need for increased education, with an emphasis on improving self-care. Furthermore, the presence of low self-confidence was shown to negatively impact the self-care behaviors among individuals with CHF [9],[24]. It is imperative to consider the role and factors (age, gender, marital status, education, and comorbidities) which influence self-confidence in the health care provider's effort to reduce the readmission rates among patients with CHF. Therefore, more time devoted to the psychosocial and psychological aspects when providing education on CHF maybe warranted by health care providers. The results of this study will be used to provide insight to staff members of the hospital regarding the learning needs of patients with CHF. Participating staff members will include registered nurses, nursing managers, nursing administration and physicians. Evidence-based recommendations on future management and education of CHF will be provided.

Specific areas of self-care that would benefit from improved CHF teaching are sodium intake, physical activity, fluid restriction, monitoring daily weights, symptom recognition, and symptom management. Effective education about self-care focuses on providing patients with the skills they need for successful management of their disease, not simply the knowledge [22]. Furthermore, identifying factors associated with CHF self-care and knowledge levels could be of high practical relevance for health-care providers. This stage of identifying factors serves as the first step in determining what patient characteristics need to be targeted in patient teaching [62].

## 5. Conclusion

The findings of this project recognize the crucial need for improved disease management among patients with CHF. Although the population demonstrated adequate knowledge of their disease process in some key areas, poor self-care behaviors were identified among most individuals and it was evident that a significant knowledge deficit was present.

Improving self-care behaviors can be obtained through effective screening and by initiating an active engagement between health care teams and the patient. Achieving positive health outcomes among CHF patients is attainable with the use of care models that foster a multidisciplinary approach. The use of CHF care models and disease management programs have been shown to improve self-care capabilities and improve knowledge.
